# Crowdfunding science

**DOI:** 10.1186/s13059-019-1864-3

**Published:** 2019-11-25

**Authors:** Melissa A. Wilson

**Affiliations:** 1 Center for Evolution and Medicine, Arizona State University, Tempe, AZ 85282 USA; 2 School of Life Sciences, Arizona State University, Tempe, AZ 85282 USA; 3 The Biodesign Institute, Arizona State University, PO Box 874501, Tempe, AZ 85287-4501 USA

After trying, and failing, multiple times to secure traditional funding to our genomics work in the Gila monster, *Heloderma suspectum*, we turned to crowdfunding. With the support (thank you!!) of 173 individual backers, we successfully crowdfunded to generate a reference genome for the charismatic black and orange Gila monster [1]. Crowdfunding was a long and involved experience. Through crowdfunding, I built new collaborations, developed an increased network of connections, and renewed my hope about the future of science funding. What I am sharing here is the learned experience of having run a successful campaign with the support and advice of many others.

I would be remiss if I did not begin by telling you about the Gila monster, a wonderfully beaded unique desert reptile. Their saliva is venomous and has been used to develop treatments for type II diabetes. Although Gila monsters live in the desert now, their ancestors lived in tropical rainforests, so they have several unique physiological adaptations to the hot, dry climate. They are protected in the state of Arizona, but their habitats are being encroached on, and they are difficult to re-home. And for the biggest nerds out there, they have a really cool sex chromosome system that is just the opposite of ours (female Gila monsters have one large and one small sex chromosome, so the eggs determine the sex of the offspring, not the sperm, like mammals). Yet at the time we started, there was no genome sequence for the Gila monster to study the molecular basis of these really fantastic features. I had recently started my lab and position at Arizona State University and wrote several grant applications to fund this work.

## Dollars and sense

There is an ongoing debate, and data investigation, into the uneven distribution of grant dollars and the impact it has. A study of the scientific impact of research funded by the Natural Sciences and Engineering Research Council of Canada (NSERC), focusing on individual researchers from three fields, concluded that impact per dollar generally decreased with increasing grant size [2]. Similarly, a study of funding allocated by the National Institute of General Medical Sciences (NIGMS) from the National Institutes of Health reported that past $700,000 in annual direct cost to the investigator per year, productivity plateaus [3]. Productivity was measured using a combination of publications and citations in both articles. Funding rates across agencies are historically low, and these studies show that exceedingly large grants are inefficient. Large grants, though perhaps not exceedingly large grants, will continue to be needed to fund many areas of research, especially including support for personnel. In contrast, crowdfunding has the potential to support small projects effectively.

Crowdfunding is not a replacement or even a competition to typical funding mechanisms, but a complement. Most crowdfunding is quite small, with a median of $3500 funded per project on one platform, Experiment.com [4]. The scale and scope of research questions that can be asked with crowdfunding are different from larger funded projects, but then, I would argue the audience is also different. When I decided to run a crowdfunding campaign to fund our work, I was an early career researcher, I had not been awarded funding from any major funding body, and we had been denied multiple times. For our project, I also had funding for personnel, and had collected samples, but needed funding for particular components of the research. The project was primed for crowdfunding. However, in the same way as there is a lot to learn that can help in crafting better grant proposals, there are many things to learn and prepare for when embarking on crowdfunding, and a lot of advice out there [5].

## Explore platforms

There are many different platforms you can use for crowdfunding, and they all come with different benefits and drawbacks [4]. Visibility is one consideration when choosing a platform. Some are explicitly for science, and some are not. In the cases where a platform is designed specifically for science projects, it may already have an audience of donors who are going there to donate to science-related projects. Some are affiliated with an institution, which could lend credibility to your research project, depending on the audience. Some follow an all-or-nothing funding model, where you either make your goal entirely, and you get all the donated funds, or you receive nothing if you do not make the goal. In contrast, others will let you keep whatever portion you raise. Some platforms limit the amount of money you can request (e.g., an all-or-nothing platform may limit your total “ask” amount, partially to assist with a higher success rate). And finally, you should explore platform fees, both what the platform itself charges (generally 3–10% of the funds you have raised) and any other charges you might receive when you move the money to your account. While I am not a tax attorney, so seek out your own advice, I learned that in some instances, if you take the money directly to your personal account, then the money may count as income, and so be taxable. The funds raised could also be donated through a University donation account, but then your institution may take a percentage cut (e.g., another 5% on top of the platform fees).

## Identify your pitch

Similar to another grant application, you need to prepare your significance and innovation, or intellectual merit and broader impacts. First, when it comes to building your platform page, then when advertising your project, you will need information for the audience about your project. Why should they care about your project? What is it about the research you are doing that they should buy into? What are the specific questions you will answer if funded? What is the funding going to make possible? Most importantly, these need to be scientifically accurate AND broadly accessible. Rather than writing to a scientific review board, you are writing for the general public. And, spoiler alert, the general public is remarkably smart and engaged with topics they care about. So, do not lose content. You can maintain accuracy while taking out jargon.

## Generate media content

Before you get started, you will want to make sure you have plenty of content about your project to share. You know and care about your research, and you learned this through a long process. The people who you are going to be asking to fund your research are unlikely to have the same depth of a relationship with the project. So, to accompany your pitch (see above), you will want to have content ready to share. Making a short video to explain who you are and why this research is important (no more than 3 min) can be a big step in personalizing the project and in introducing you to the audience. You should have a list of citations of primary research and secondary sources, like news articles or popular science articles, lined up, each with the citation and a short (think tweet-able) blurb about them. And, finally, I recommend to have a set of images that can be shared via social media about the project. These can be you in the field or in the lab. These can be about your study system, or about the people it will impact. Each should have a caption explaining the photo, giving credit if not your own photo, and could even accompany the citations you have collected.

## Build a network

Unlike a government or foundation funding agency, in the case of crowdfunding, your audience is the world (hello, world). Before your funding campaign goes live, you should be prepared with the set of potentially interested groups to advertise to. If you study a disease, then research the online presence, forums, and social media accounts related to that disease. If you study a particular species, prepare a list of contacts across groups that support conservation or research of that species. You may want to develop a social media presence for your project. Think of this as an account that you can use to interact with your donors and share content in a way that is separate from your personal accounts.

While you may want to have an account solely for your project, your network and donors will be deeply personal. Unlike a grant, where you are asking a remote panel to evaluate your ideas and fund you, one of the largest groups of people who will support your crowdfunding campaign are your colleagues, collaborators, friends, and family. This was uncomfortable for me, largely because of the huge pressure. To have people I know, who I interact with, donate to my research, made it all so much more real and personal.

## Allocate time

You will spend a substantial amount of time on a successful campaign throughout the whole process, from build-up, to campaign, to follow-up. You will need to reserve time to prepare all the content needed for starting the campaign (let us say, 40–80 h of work, spread over several months). Then, I highly recommend you block out time (at least an hour) *every day* that the campaign is live to track progress, respond to inquiries, share the content you prepared, and continue with your asks and advertising to different groups. Most campaigns run 30 days. You will need to keep track of any rewards you may have offered, who they will go to, and when you will be sending them out. You should write a prompt “thank-you” note to every donor—having a template prepared ahead of time is a good idea. You can also reach out to enthusiastic donors to help spread the word about your project to their friends, family, and professional networks.

## Costs and benefits of crowdfunding

In my experience, crowdfunding includes a lot of benefits. Perhaps, one of the biggest benefits is actively building a community of interest in the project. I built and maintain connections as a function of running the crowdfunding campaign, and I still get to hear exciting updates from these people. I think it was tremendously valuable to have a direct connection with the donors and be able to answer their questions directly about the research project. For me, especially after having so many failures, I cannot overstate the boost to morale of having the project funded. The relatively high success rates of crowdfunding were one driver for me to try it out.

That said, there are several drawbacks to crowdfunding. First, it can be quite uncomfortable to ask for money, over and over again. With a grant, there is a single submission (or maybe a couple of rounds of whittling down), but with crowdfunding, you are asking every single day of the campaign for people to support you. Second, the platform we used is “all-or-nothing”, and that added a level of stress and urgency to the project. Finally, compared to more traditional funding mechanisms, it is an equal or greater amount of work for a much lower amount of funding. In our case, it funded what we needed, but I do not see how crowdfunding could sustain a lab that needed to pay personnel. That is just not the goal of crowdfunding.

## Should you crowdfund?

So, perhaps the last question is, should you crowdfund. Well, that depends. If you can answer yes to all of these, then I think you are starting off on the right foot.

- Do you have a specific question?
- Can you persuade people to care about your question?
- Can you address your question with a small amount of money?
- Do you have time to devote to promotion of the project?
- Can you ask people for money, over and over?

I am now a few years removed from our crowdfunding campaign, and I still believe all of the effort was worth it. The funding is amazing, and the experience still blows me away—that so many people believed in our project and our capacity. Because of those funds, we have gotten to do what we set out to do; we now have a reference genome for one of the most interesting animals on the planet (yes, those are fighting words). Because of the network building, I was additionally able to support additional analyses to study Gila monster sex chromosomes! Because of the people I met due to the crowdfunding campaign, I got to learn about a nest of baby Gila monsters that was dug up while they were hatching (!!), collaborate with new technology companies, and connect with Gila monster researchers and enthusiasts from far and wide. Surely, this will not happen with every crowdfunding campaign, but it is possible, unlike grants, which are stuck behind closed doors and anonymous peer review. The public judged us, and joined us.

Crowdfunding has the capacity to open the door to even more scientists and project ideas, especially those that are not fundable through traditional methods. It can help a trainee finish the last bit of a project, generate preliminary data for a new area, or fund an important reference for your ongoing research. Crowdfunding is also more than money for a project. Crowdfunding forces us to step back from the lab and place our research in a broader context, in a very concrete way. It provides the opportunity to publicly share our passion and enthusiasm for our research.
